# The interpretation-use argument– the essential ingredient for high quality assessment design and validation

**DOI:** 10.1007/s10459-024-10392-6

**Published:** 2024-11-26

**Authors:** Jacqueline Raymond, David Wei Dai, Sue McAllister

**Affiliations:** 1 School of Health Sciences, Faculty of Medicine and Health, The University of Sydney, Sydney, Australia; 2 UCL Institute of Education, University College London, London, UK; 3 Faculty of Health, University of Canberra, Canberra, Australia

**Keywords:** Validity, Argument-based approach, Inferences, Workplace-based assessment, Clinical competence

## Abstract

There is increasing interest in health professions education (HPE) in applying argument-based validity approaches, such as Kane’s, to assessment design. The critical first step in employing Kane’s approach is to specify the interpretation-use argument (IUA). However, in the HPE literature, this step is often poorly articulated. This article provides guidance on developing the IUA using a worked example involving a workplace performance assessment tool. In developing the IUA, we have drawn inspiration from approaches used in the discipline of language assessment to situate the inferences, warrants and assumptions in the context of the assessment tool. The worked example makes use of Toulmin’s model of informal logic/argumentation as a framework to structure the IUA and presents Toulmin diagrams for each inference such that the reader can connect the argument chain together. We also present several lessons learned so the reader can understand the issues we grappled with in developing the IUA. A well laid out IUA allows the argument to be critiqued by others and provides a framework to guide collection of validity evidence, and therefore is an essential ingredient in the work of assessment design and validation.

## Introduction

*“If the interpretive argument is not specified clearly*,* there is potential for mischief.”* (Kane, 2004, p. 165).

Within health professions education (HPE) there has been increased interest in modern conceptions of validity, in particular argument-based approaches. These approaches focus on curating evidence, numerical or otherwise, to defend decisions based on an assessment outcome (Cook et al., 2015). One such approach is that espoused by Kane (Kane, 2013) and involves two key steps. First, establishing a reasoned argument specifying the inferences and assumptions inherent in the proposed interpretation and use of assessment outcomes. This is the interpretation-use argument (IUA). Second, collating evidence to establish the plausibility of the claims made in the IUA. This is the validity argument (VA). Importantly, these two steps work together; the IUA provides the framework for the VA and the VA provides evidence that the IUA is coherent and plausible.

While many examples in the HPE literature use Kane’s approach, few report adequately on the first step of specifying the IUA (Kinnear et al., 2022). Instead, selective pieces of validity evidence are reported without a framework that permits scrutiny of the argument to justify the intended interpretation and use of the assessment outcome (Kinnear et al., 2022). Providing validity evidence without first outlining an IUA defeats the purpose of argument-based validity for two reasons. First, the valid use of the assessment is fundamentally undermined if the intended interpretation and use of the assessment outcome is not clearly specified. Second, if it is not clear what assumptions have been identified, or whether these are the most critical and relevant, we cannot be confident that it is valid to use the assessment for the purpose intended. This is the kind of mischief all assessment developers wish to avoid!

This paper provides guidance to HPE assessment developers through sharing a worked example of an IUA for a performance-based assessment currently in development. In sections to follow, we provide an overview of Kane’s argument-based approach with a focus on the IUA; we outline how assessment validation in HPE can draw from the discipline of language assessment to expand and strengthen validation frameworks; we use Toulmin’s model of informal logic/argumentation (Toulmin, 2003) as a strategy to structure the IUA; and finally we outline the challenges experienced and lessons learned so far in using Kane’s approach to argument-based validity. Our goal is to make the process of applying the principles of Kane’s approach more explicit and thereby support HPE assessment developers to maximise the flexibility and innovations this approach can offer (Cook et al., 2015).

## Argument-based approach to validation

Contemporary argument-based approaches consider validity to be the robustness with which one can interpret and use assessment outcomes, and validation as the process of generating an argument to support the proposed interpretation and use (AERA, APA & NCME, 2014). A range of argument-based approaches exist (see Lavery et al., 2020 for a brief overview of many of these). For example, Schilling and Hill (2007) prescribe three general categories of assumptions and inferences to be considered when developing a validity argument (elemental, structural and ecological); Bachman (2005) refers to an ‘assessment use argument’ with four types of warrants to guide evidence collection; and Sireci (2013) proposes using the five sources of evidence in the Standards for Educational and Psychological Testing (AERA, APA & NCME, 2014) to guide a validity argument. Kane’s approach (Kane, 2013) develops a chain of inferences for the interpretation and use of assessment outcomes which provides guidance for collecting validity evidence. This approach has garnered considerable attention in the HPE literature, and while its flexibility has been criticised for being impractical (Schilling, 2004), it is well suited to a wider range of assessment types with varying complexity such as those we use in HPE (Cook et al., 2015). It is due to this flexibility along with its familiarity to HPE scholars, that this paper focuses on Kane’s approach.

**Fig. 1:**
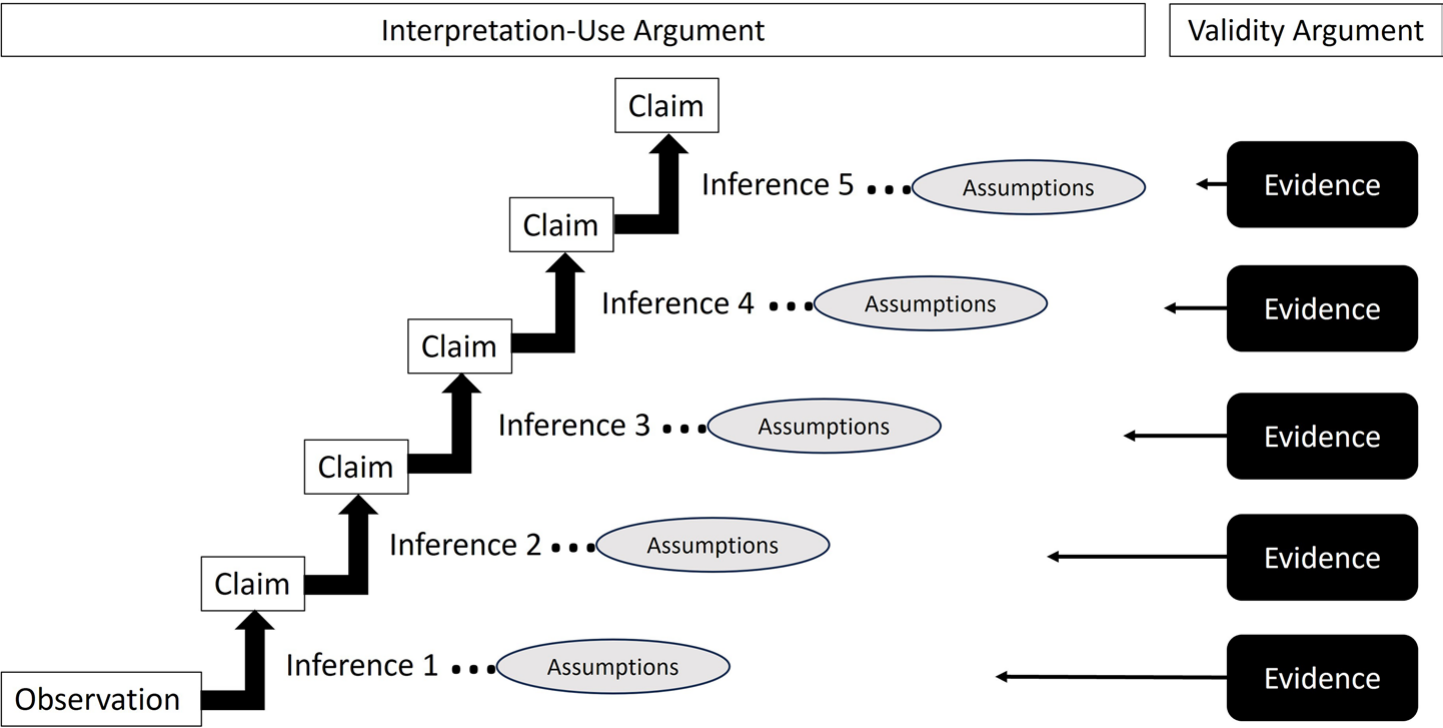
The connection between the interpretation-use argument and the validity argument. The interpretation-use argument lays out a coherent sequence of claims and the assumptions inherent in the claims being made. The validity argument provides evidence to support the assumptions and therefore substantiate the claims being made. While the figure presents a distinction between the two arguments, in practice they are intertwined and considered together

Kane states that the argument-based approach “is basically quite simple. First, state the claims that are being made in a proposed interpretation or use (the IUA), and second, evaluate these claims (the VA).” (Kane, 2013, p. 9). However, while the concept seems simple, operationalizing this framework is not a straightforward process (Chapelle, 2012), something that even Kane himself recognises (Kane, 2012). First, the IUA must present as logically sequenced and coherent, and second, the right theoretical and empirical pieces of evidence need to be synthesised into an argument (the VA) for the plausibility of the IUA (Kane, 2013). Furthermore, while the approach appears as two steps, the two components, the IUA and the VA, are intertwined (Kane, 2013) and very much rely on each other (Fig. 1). The VA is needed to establish the plausibility of the IUA, and the IUA is needed to provide the framework for the VA. Without the framework there is “*potential for mischief*” (Kane, 2004, p. 165) as it is not clear what the validity evidence is arguing for.

## The interpretation-use argument

To validate a proposed interpretation and use of an assessment outcome, one must be clear about what is being claimed and then detail a plausible argument leading from observed performances to the claims being made based on these performances (Kane, 2013). The IUA lays out this argument by systematically describing the chain of inferences being claimed and the assumptions inherent in these. Systematically laying out the IUA, allows for gaps to be identified or the plausibility of the argument overall to be challenged (Knoch & Chapelle, 2018). It also provides clear guidance to a potential user on whether the assessment outcomes match their intended interpretation and use. Finally, it also provides a rationale for the evidence to be collected to evaluate the claims being made.

Kane’s approach to constructing the IUA is not prescriptive. When explaining how an IUA could be framed, Kane typically identifies four inferences in the chain from assessment outcome to use of that outcome– scoring, generalization, extrapolation and utilization. These same four inferences are consistently used in the examples in the HPE literature that apply Kane’s approach. However, Kane states that we are not bound to these four inferences; “they are intended as examples and not as a checklist” (Kane, 2013, p. 10). Instead, the number and type of inferences in a chain should be those required to provide a reasoned argument specific to the intended use of the assessment outcome. Different numbers and types of inferences and assumptions will be required for different purposes and assessment types. Looking outside of the HPE literature, we can find examples where this is the case (e.g. Chapelle et al., 2011; Hopster-den Otter et al., 2019; Fechter et al., 2021).

## Problematic reporting of the interpretation-use argument in health professions education literature

Kinnear et al. (2022) highlight that there are limited examples in the HPE literature where the IUA is presented in a way that can be scrutinized and debated. Some of the problematic representations of the IUA include: using the four inferences from Kane’s examples but without arguing why these are the most appropriate for the assessment under consideration (e.g. Wijnen-Meijer et al., 2013; Colbert-Getz et al., 2015; Hatala et al., 2015); not reporting assumptions (e.g. Gadbury-Amyot et al., 2014; Colbert-Getz et al., 2015); and reporting only a single inference which is not situated in a reasoned argument leading from assessment outcome to interpretation and use (e.g. Colbert-Getz et al., 2015; Sheppard et al., 2023). Failing to systematically lay out a complete IUA limits the ability of potential assessment users to critically assess the robustness of the validity argument (Chapelle, 2012; Kinnear et al., 2022). Furthermore, this may lead to a focus on presenting validity evidence that is at hand, easily collected, or collected without purpose (Cook & Hatala, 2016; Kinnear et al., 2022), with unknown connection to the intended interpretation and use of an assessment outcome - sources of mischief all assessment developers would want to avoid.

## What can we learn about the interpretation-use argument from other disciplines such as language assessment?

The quality of HPE research has frequently benefited through what has been learned in other disciplines (Schuwirth & van der Vleuten, 2011). The discipline of language assessment has a well-established literature on validation and the application of Kane’s validity framework (Dai et al., 2024). This discipline’s focus on assessment of communication skills is also closely aligned with topics familiar and of interest to HPE researchers such as clinical communication.

Language assessment focusses on language users’ ability to use language for effective communication. Because language ability is considered crucial for high-stakes decisions such as admissions to university courses, international migration, and citizenship, there has been continuing strong demand for standardised, validated, large-scale language assessments, for example, Test of English as a Foreign Language (TOEFL) and International English Language Testing System (IELTS). The commercial nature of language assessment companies also requires each large-scale language test to produce sound, stakeholder-accountable validity arguments for their test products, which has led to ongoing multi-million-dollar validation projects (see for example, Chapelle et al., 2011 for TOEFL). These industry-led validation practices have also reflexively informed theory construction and have prompted further specification of validation frameworks in language assessment, with Kane’s argument-based framework being one of them.

Language assessment researchers have had a long history of iterative engagement with Kane’s argument-based approach (Chapelle et al., 2011; Chapelle & Voss, 2021) which has elevated the sophistication with which the IUA is presented. For example, current argument-based validation endeavours in language assessment routinely include a domain description inference before the scoring inference, an explanation inference between the generalisation and extrapolation inferences, and a utilization and a consequences inference after the extrapolation inference (Chapelle et al., 2011; Knoch & Macqueen, 2019).

The *domain description* inference originates from Kane’s writing on the concept of test domain (Kane, 2013). Language assessment researchers have noted that the domain of a test needs to be clearly defined to ensure the test is evaluating performances that are relevant to the domain. Some representative assumptions underlying the domain description inference are that the assessment tasks (1) represent activities in the test domain, and (2) offer a comprehensive coverage of the test domain. The evidence for these assumptions is commonly gathered by a domain or needs analysis at the outset of a test development project (see Dai, 2023 for an example).

The *explanation inference* is based on Kane’s argument that it is problematic to assume that an underlying trait or construct explains observed behaviours (Kane, 2013). This can be particularly challenging to ascertain when the underlying trait is related to language use and communication because of the complexities involved in describing language use as an assessment construct (Dai, 2024). Language assessment researchers therefore see the necessity to separately gather evidence to evaluate whether the outcomes can be attributed to the theoretical trait the assessment was designed to measure. Commonly identified assumptions underlying the explanation inference are (1) the skills required to complete the assessment tasks are congruent with the ones identified in relevant theories, (2) the structure of the test (e.g., how different skills are assessed and defined) is consistent with theoretical frameworks of the underlying construct, and (3) test performances on the instrument being developed align with performances on assessments that claim to measure the same or similar constructs.

Finally, the *utilization* and *consequences inferences* in language assessment, are further elaborations and specifications of what Kane originally called implications. Language assessment researchers noted that when argument-based validation researchers discuss implications, they frequently conflate two types of implications: (1) how decisions are made based on test results, and (2) whether test results are beneficial to stakeholders who use the test. These two aspects of implications have distinctive, independent warrants and assumptions, especially since the latter can only be ascertained when the test has become operational (Knoch & Chapelle, 2018). Therefore, language assessment researchers have separated the broad, generic implication inference into the utilisation and the consequences inference, each with their own warrants and assumptions.

The utilisation inference needs to be considered during the test construction phase. The warrant behind this inference is that the decisions (e.g., pass or fail) can made based on test results and communicated to different test-using stakeholder groups, such as test takers and organisations that make decisions based on test results. Common assumptions behind the utilisation inference are (1) the test and its accompanying rubric can differentiate test takers into meaningful and appropriate levels that facilitate decision making, and (2) the cut-scores used for decision-making are defensible.

The consequences inference is concerned with whether the test use is beneficial to test users and whether it can generate long-term positive influence. The warrant behind the consequences inference is that the test generates positive outcomes for relevant test-using stakeholders. Commonly identified assumptions are (1) the test-takers that have passed the assessment can function satisfactorily in the test domain, (2) the assessment provides a positive model of the assessed trait for test-users, and (3) test scores and assessment feedback contribute positively to teaching, learning and assessment.

## Worked example

### Context for our worked example

Accredited exercise physiologists (AEP) are Australian allied health professionals who graduate as autonomous practitioners. Exercise physiology (EP) students participate in multiple clinical placements of varying duration to develop their professional competence. When on placement, students are immersed in the work environment and typically work closely with an AEP who supports learning through integrating the student into all aspects of service delivery, providing feedback, and assessing performance.

The performance-based assessment tool under development and used in the worked example is based on a holistic and integrated conception of competence where competent performance is underpinned by combinations of knowledge, skills and capacities (Heywood et al., 1992). The design of the tool includes the following content and processes. Six broad and interrelated units of competency are described that relate to competent EP practice. Each unit of competency includes 2–4 elements (Raymond et al., 2020). An example unit of competency is shown in Table 1. The placement educator forms a judgment of the student’s performance against each element based on a synthesis of observations made across the placement. This judgement is recorded along a visual analogue scale (VAS). The VAS includes three descriptors describing the decreasing level of supervision and support required to competently perform the element. The assessment tool supports placement educators to make a judgment by including detailed profiles of performance for each of the three descriptors along the VAS. These profiles are designed to provide a frame of reference to support making a judgment.

**Table 1:**
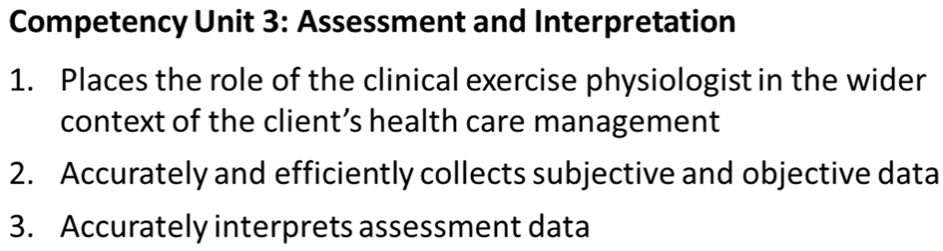
Competency unit 3 which contains 3 elements (Raymond et al., 2020)

Tool design included consideration of Tavares and colleagues’ (2020) “compatibility principle” which recommends considering different philosophical positions, ontologies, epistemologies and how claims made based on an assessment outcome can be justified. While it is beyond the scope of this manuscript to fully lay out the philosophical position influencing the design and development of the assessment tool, a brief overview is warranted to illustrate coherence with our approach to validation. A post-positivist philosophical position informed the design and development of the assessment tool. Post-positivism acknowledges bias in observations and while a single objective truth is desired, it can never really be achieved (Young & Ryan, 2020). Aligned with this position, we defined competence as a collection of latent traits within an individual (Heywood et al., 1992). This competence is applied by the individual to the workplace and is, in turn, influenced by their personal circumstances and the affordances and constraints of that workplace (Rethans et al., 2002). Competence is not directly measurable but can be inferred from observations of performance in a workplace context. In our assessment tool, these observations are represented as a judgment on the VAS. The judgment is intended to be an accurate representation of these performances although it is recognised there are many assumptions underlying this.

### Laying out the argument– toulmin’s model of argumentation

Kinnear and colleagues (2022) contend that when applying argument-based approaches to validation, such as Kane’s approach, HPE scholars need to present the structure of their arguments more explicitly so that they can be open to analysis and evaluation. However, how one goes about structuring “the network of inferences and assumptions” (Kane, 2013) such that it can be open to scrutiny is not intuitive. We found that using Toulmin’s model of informal logic/argumentation (Toulmin, 2003) as a framework to structure our IUA assisted us to make our inferences and assumptions explicit.

Informal logic, an argumentation theory, involves real world arguments where claims are made, assumptions are never watertight, and the strength of inferences depends on the degree of empirical support (Kane, 2004). Informal logic therefore lends itself well to Kane’s approach because interpretive arguments typically involve inferences and assumptions when moving from observed performances to conclusions made about these performances (Kane, 2004). Informal logic also lends itself well to the often-complex HPE validity arguments, providing a means for clearly structuring these in a way that they can be scrutinised (Kinnear et al., 2022).

At the core of the Toulmin’s model of argumentation is a *claim* that is based on a *datum*, or piece of information that leads to the claim, in accordance with a *warrant*. The warrant is a hypothetical bridging statement between the datum and the claim which makes the claim legitimate (Toulmin, 2003). Other components include *backings* for the warrant, or sources of evidence that make the assumptions inherent in the warrant reasonable, *qualifiers*, which speak to the strength of the warrant, and *rebuttals*, which are the conditions under which the warrant might not be true. To explain, Toulmin (2003) used the example that Harry is a British subject (the claim) based on the fact Harry was born in Bermuda (the datum) since a person born in Bermuda will generally be a British subject (the warrant). Backing for this warrant could take the form of laws covering the nationality of those born in British colonies (Toulmin, 2003). The warrant is not necessarily absolute, so requires a qualifier– in this case “so, presumably” (Toulmin, 2003). There are also circumstances under which the warrant might not hold up such as Harry becoming an American citizen (the rebuttal) (Toulmin, 2003).

Our worked example is naturally more complex than the example provided by Toulmin (2003). There are multiple inferences and assumptions and these need to be organised in a coherent way such that the IUA can be scrutinised. Therefore, multiple Toulmin models were used and connected into a network to illustrate the coherence of the overall argument. To achieve the connection, we outlined a Toulmin diagram for each inference, with the claim from one inference becoming the datum for the next thus connecting the inferences into a chain.

The Toulmin models presented in the worked example that follows use the terminology defined in Table 2. However, it is important to recognise there can be variations in terms and how these are used by others in their argument structures. For example, the warrants proposed by Wijnen-Meijer and colleagues (2013) might be considered equivalent to backings in Toulmin’s model. Furthermore, for a given interpretation and use, an assessment developer might generate a different series of arguments where components that we consider to be assumptions might be a warrant or a claim, or claims might be ordered differently. Kane’s argument-based approach to validation supports this flexibility.

**Table 2:** Definitions of the components of Toulmin’s model of argumentation (Toulmin, 2003)

Components	Definitions drawn from Toulmin (2003)
Claim	An assertion; a position or a conclusion that will be argued for (p. 90)
Datum	The facts that are the foundation of for the claim (p. 90). Sometimes also called “Grounds”, i.e. the grounds for the claim.
Warrant	A bridge to show how the datum gives rise to the claim (p. 91). The warrant justifies the leap from datum to claim.
Backing	Evidence which strengthens the warrant (p. 96). The evidence targets the assumptions in the argument.
Qualifier	Words used to indicate the strength of the warrant in legitimising the leap from datum to claim. (p. 93) For example, words such as “probably” and “presumably”, soften the claim so that it is not absolute.
Rebuttal	Conditions under which the claim might be invalid (p. 94)

### The IUA for a performance assessment tool

#### Proposed interpretation and use of the assessment outcome

The placement educator’s judgment of a student’s performance is an accurate characterisation of the student’s clinical competence in the AEP professional workplace (*interpretation*). The placement educator’s judgment can be used to make decisions about advancement towards and readiness for entry-level practice (*use*).

#### Chain of inferences

We developed the chain of inferences in the IUA and identified warrants and assumptions through a series of team meetings. This iterative process of building a plausible IUA through continually challenging the chain of inferences and underlying warrants and assumptions is standard practice in the discipline of language assessment and ensures rigour in the validation process. We remained open to developing as many inferences as needed and were informed by those found in language assessment validation frameworks (Dai et al., 2024).

First, the domain description inference was considered relevant to our context, although we concluded it could not be used in the same way it is used in language assessment. In our context, we are not creating a standardised test independent of the workplace setting. Instead, this assessment is occurring in an existing environment (an AEP workplace) that is unpredictable and not overly prescribed. Furthermore, the extent to which students can legitimately participate varies and is influenced by a range of factors, including workplace culture (Dornan et al., 2007; Boor et al., 2008), and placement structures (Bernabeo et al., 2011; O’Leary & Cantillon, 2020). Therefore, the primary consideration is whether the environment in which the assessment is being conducted creates opportunities for students to perform rather than designing a standardised test with tasks that represent the activities in the test domain. Given the different contexts, we used the name ‘domain inference’ rather than ‘domain description inference’.

**Fig. 2:**
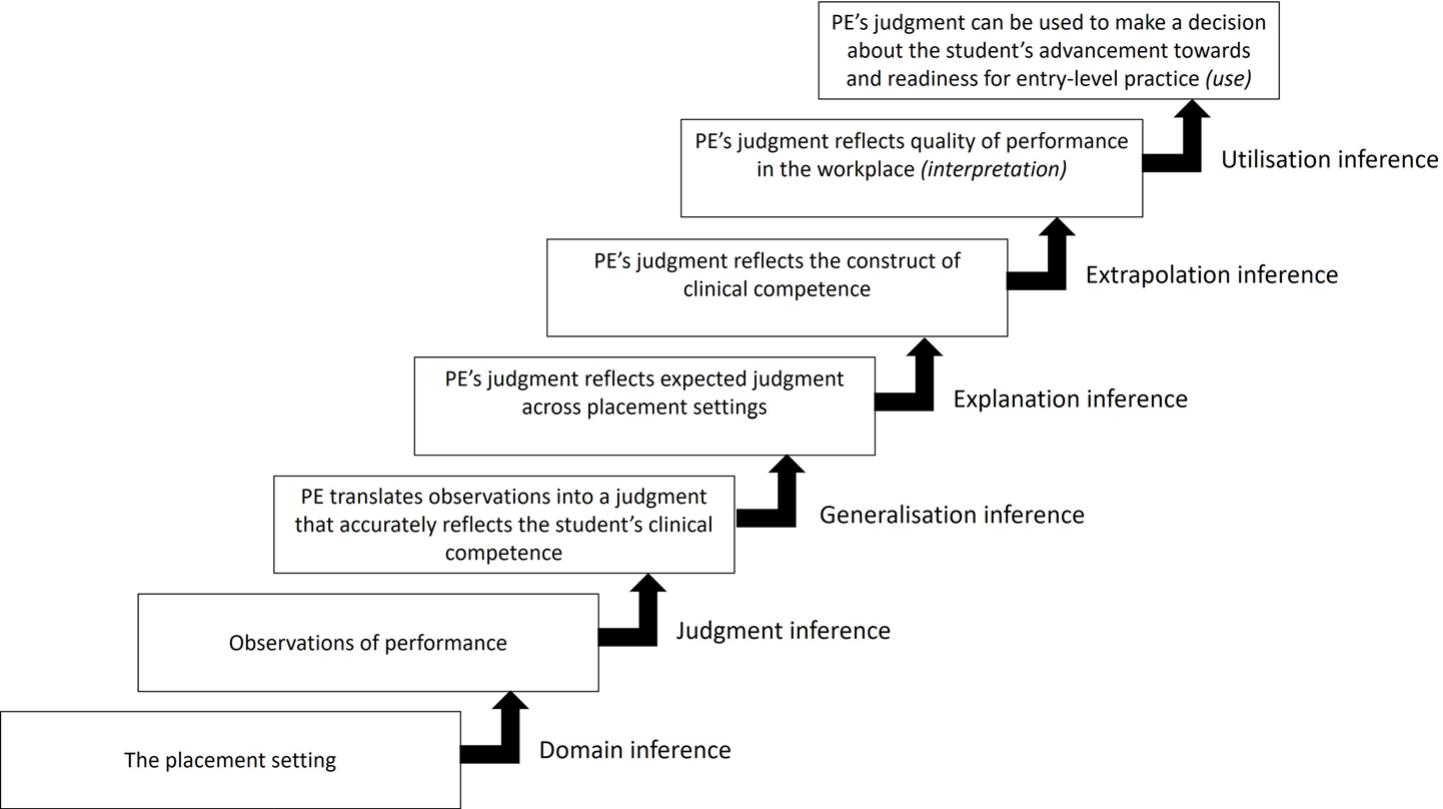
Chain of inferences in the final interpretation-use argument for the performance assessment tool in the worked example. PE: Placement Educator

Next, we included an explanation inference due to the complexity of clinical competence as a construct and to avoid the risk to validity in presuming the assessment tool provided an assessment of this construct. We also included a utilisation inference, but not a consequences inference because we are in the assessment development stage and therefore only concerned with the inferences involved in constructing the assessment. A consequences inference may be added later when the assessment tool is in full use. The inferences in the IUA also included those that would be familiar to HPE researchers, namely the scoring inference (although we refer to this as the judgment inference to reflect the assessment outcome being a judgment, not a score), the generalisation inference and the extrapolation inference. The chain of inferences in the final IUA for this worked example is shown in Fig. 2 and illustrates the linkage from performance in the placement setting through to a judgment that reflects a student’s clinical competence in the professional workplace (*interpretation*) and a decision based on that judgment.

#### Domain inference

**Fig. 3:**
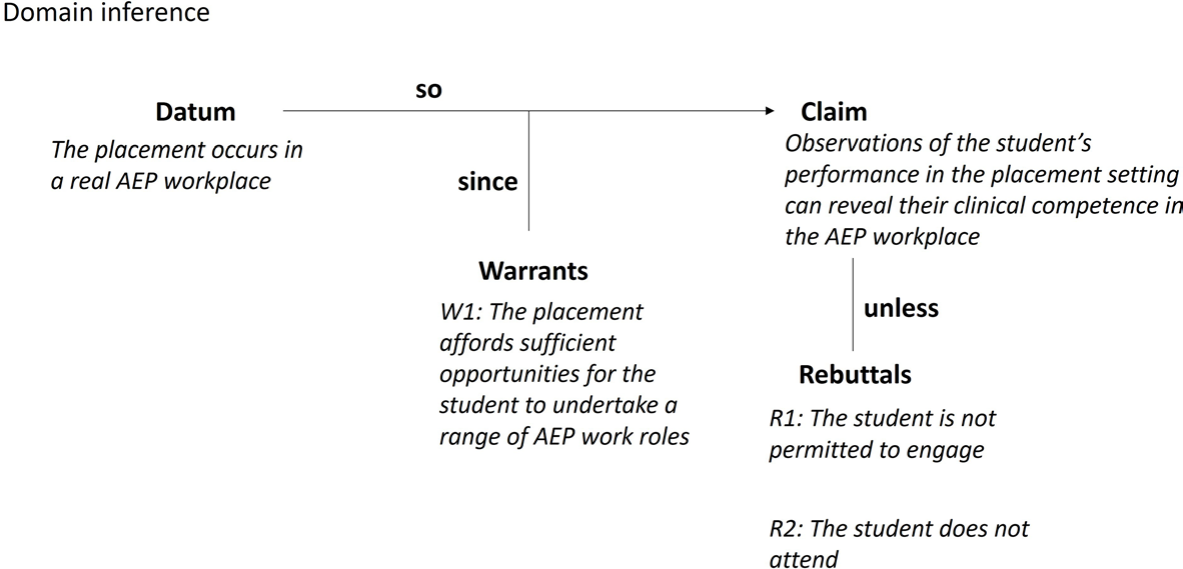
Toulmin diagram for the domain inference

The Toulmin diagram for the domain inference is shown in Fig. 3. The assessment domain is the clinical placement which is undertaken in a professional AEP workplace setting. The domain inference proposes that the observations used to make judgments about clinical competence involve performances drawn from professional workplace setting. This claim is reasonable since the placement affords sufficient opportunities to undertake a range of AEP work roles.

There are two main categories of assumptions underlying the warrant that connects the datum to the claim. First, appropriateness of the test domain i.e. are tasks performed on placement representative of the tasks in the AEP professional workplace. Second, students are afforded the opportunity to legitimately participate in the placement. The assumptions include:

- A1: The learning environment on placement affords opportunities for the student to perform across a sufficient scope of AEP work roles during their placement.
- A2: The placement provides students with sufficient opportunities to learn through participation in service delivery during their placement.
- A3: The duration of the placement is long enough to enable students to participate sufficiently to offer adequate coverage of the roles of AEPs in the professional workplace.

A potential source of backing for the warrant that would contribute to the VA includes an analysis of the placement environment. For example, a review of placement agreements, an observational study of students while on placement, and regular auditing of placement sites.

#### Judgment inference

**Fig. 4:**
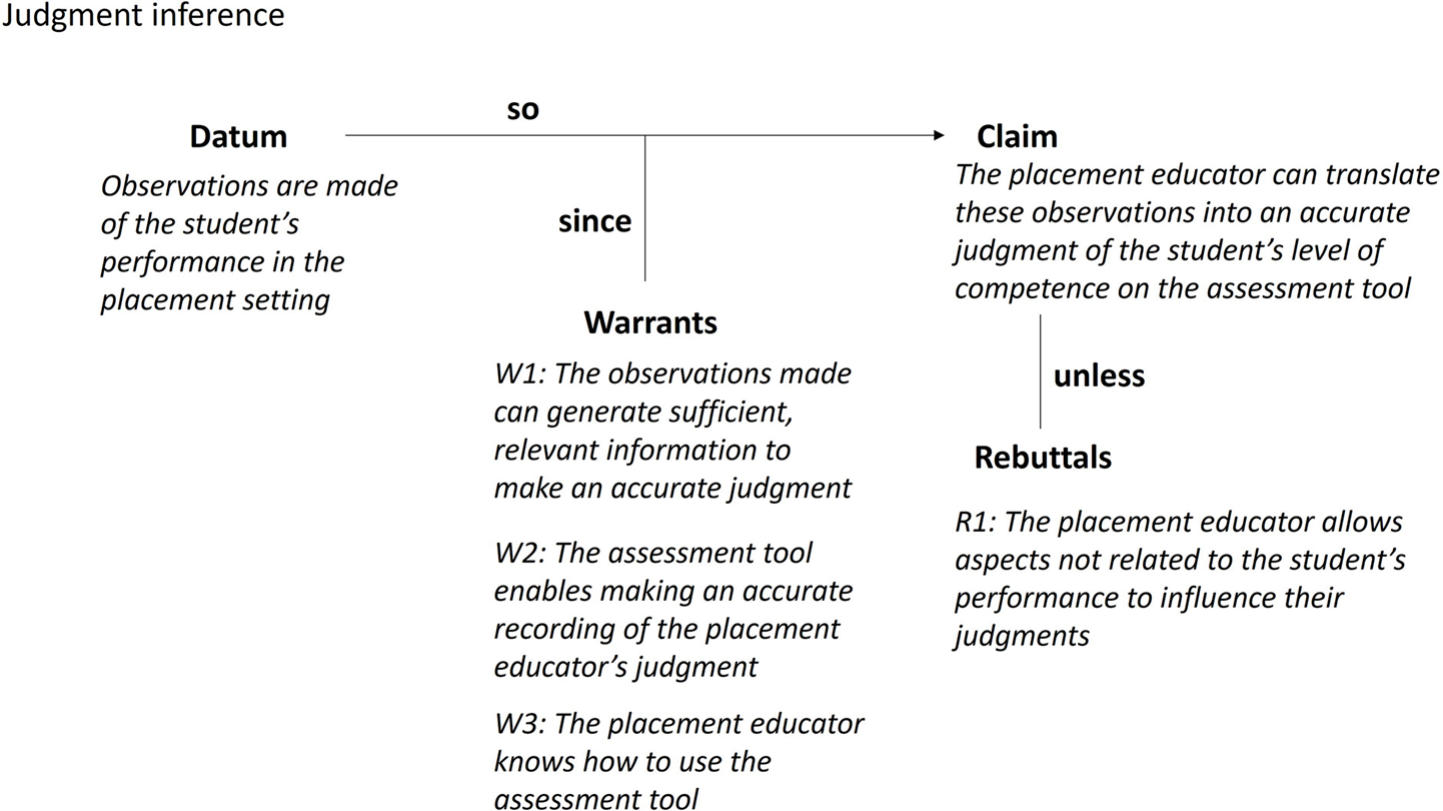
Toulmin diagram for the judgment inference

The Toulmin diagram for the judgment inference is shown in Fig. 4. We have used the term “judgment inference” (as opposed to “scoring inference”) to reflect the assessment outcome being a judgment, not a score. The judgment inference proposes that the student’s performance on placement is observed and that a placement educator can translate these observations into a judgment recorded on the assessment tool that accurately reflects the student’s level of competence. There are three warrants underlying the claim made in the judgment inference. First, that the observations permit an accurate judgment to be made; second, that the assessment tool enables an accurate recording of the judgment; and third, that the placement educator knows how to use the assessment tool to make a judgment. The assumptions underlying the three warrants include:

- A1: The placement educator actually observes student performances and uses these observations to construct their judgment.
- A2: The placement educator makes sufficient observations across the range of EP work roles such that further observations do not add new information to the judgment.
- A3: The range of performances the placement educator has observed is sufficiently representative of performance across the whole placement.
- A4: The range of performances on which the placement educator bases their judgment is sufficiently broad to ensure all aspects of the construct are observed.
- A5: The VAS used to record a judgment based on observed performance is used as intended.
- A6: The VAS used to record a judgment based on observed performance functions as intended.
- A7: The VAS on which a judgment is recorded enables placement educators to record different levels of the construct in a consistent and predictable manner.
- A8: Individual placement educators are consistent in the way they use the tool with each student.
- A9: The placement educator possesses and maintains sufficient expertise to use the assessment tool to make and record their judgements.
- A10: Clinical competence can be inferred from observations of performance.

Potential sources of backing for the warrant include an audit of the processes in place to support placement educators in using the assessment tool effectively. For example, placement educator training, the extent of ongoing monitoring to check placement educators continue to use the assessment tool effectively, and real time observations of how a placement educator makes observations and uses the assessment tool. Other potential sources of backing for the warrants come from the design and functioning of the assessment tool. These sources could include a theoretical argument about how the design features support quality judgments, and statistical analysis to identify how well the scale differentiates between performances.

#### Generalisation inference

**Fig. 5:**
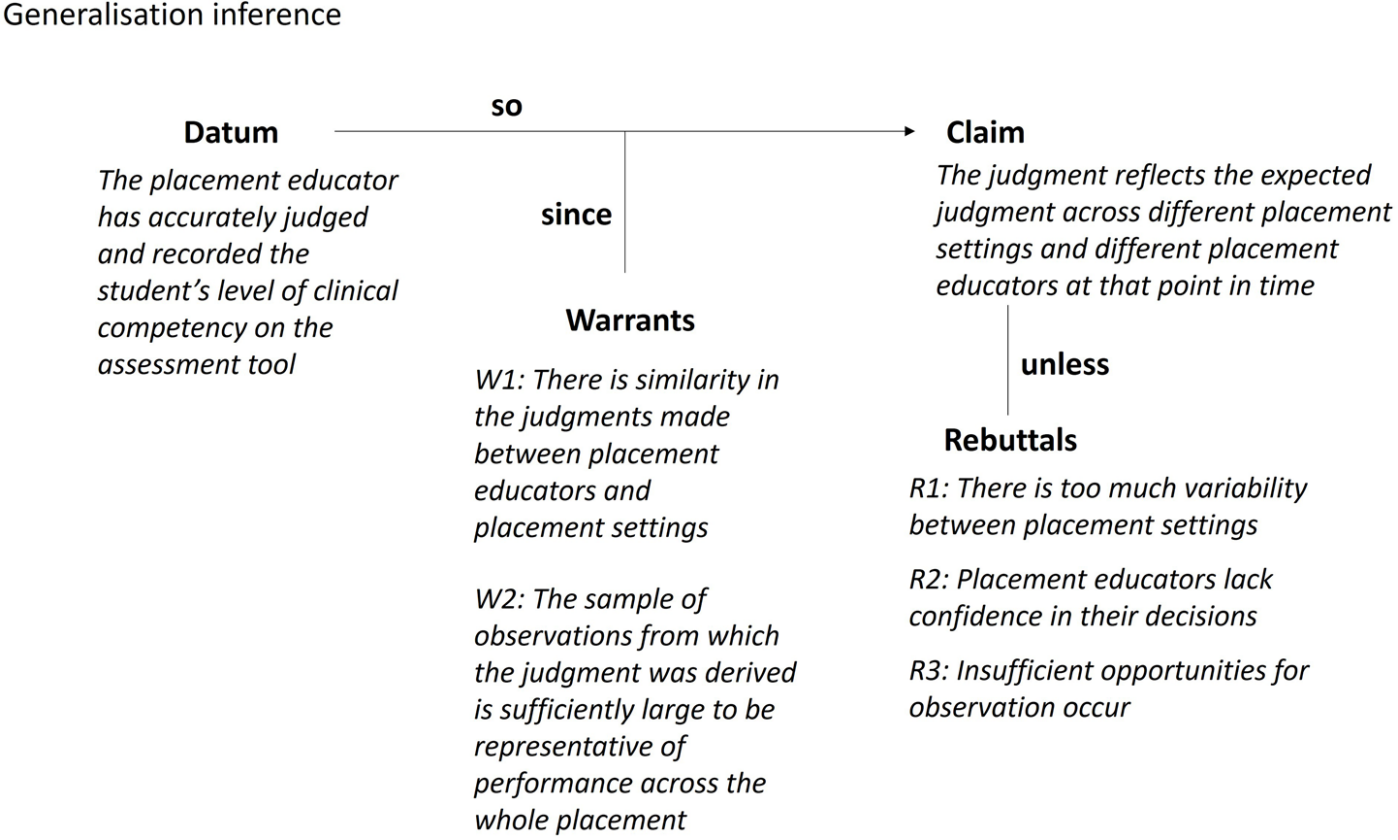
Toulmin diagram for the generalisation inference

The Toulmin diagram for the generalisation inference is shown in Fig. 5. The generalisation inference proposes that a placement educator’s judgment of a student’s performance will be consistent with other placement educators who have made the same observations of the student. The inference also proposes that judgments will be consistent if the student is observed in parallel settings. That is, the judgment would be the same regardless of which placement the student is on at that point in time.

There are two warrants underlying the claim made in the generalisation inference. First, that judgments made between placement educators in the same or different placement settings will be similar; and second, that the sample of observations used to form a judgment is sufficiently large to be representative of performance across the whole placement. The assumptions underlying the two warrants include:

- A1: Different placement educators in the same workplace at the same time make similar judgments about a student’s performance.
- A2: The same student participating in a different placement at a given time is assessed the same by a given placement educator.
- A3: The placement that the student is on is representative of all clinical placements available at that point in time.
- A4: There is reasonable transfer of competency across different pathology areas and practice areas.
- A5: The placement educator samples a large number of observations.

One source of backing for the warrants might be a theoretical argument about design features within the assessment tool that support shared mental models regarding entry-level performance and its developmental continuum. Other potential sources include conducting interviews with different placement educators in the one setting to understand how they arrived at their judgment of the student’s performance, and interviews with placement educators from a range of settings to understand how they arrive at a judgment. Statistical analysis could also be used to provide evidence for reliability and assessor agreement.

#### Explanation inference

**Fig. 6:**
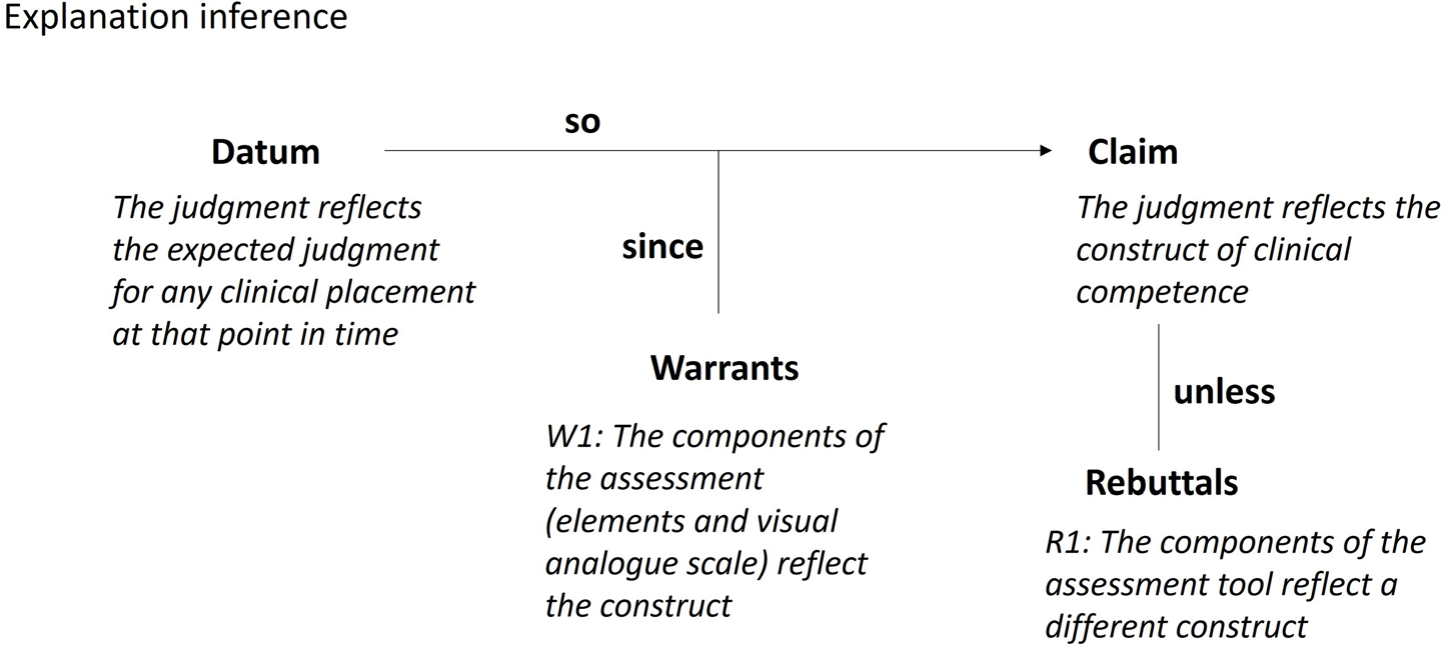
Toulmin diagram for the explanation inference

The Toulmin diagram for the explanation inference is shown in Fig. 6. The explanation inference proposes that the expected judgment over the test domain can be attributed to the construct (i.e. clinical competence) that the assessment tool was designed to record. The warrant underlying this claim is that the components of the assessment tool reflect the construct of clinical competence. The assumptions underlying the warrant include:

- A1: The behavioural descriptors along the visual analogue scale represent a theoretical model of developing expertise leading to entry level clinical competence.
- A2: Placement educators’ conceptualisation of the construct of clinical competence and its development is consistent with the theoretical model underpinning the VAS.
- A3: The units of competency and associated elements in the assessment tool reflect EP professional practice in a complete way.
- A4: The VAS and the competencies (units and elements) are sufficient for sampling a unidimensional latent construct.

Potential sources of backing for the warrant include the theoretical and empirical evidence underpinning the design of the assessment tool and an expert review of this process. Other sources of backing might include a talk aloud study where placement educators talk through what is informing their judgment, comparisons with other tests of clinical competence and a theoretical argument supporting the notion that competence can be inferred from performance.

#### Extrapolation inference

**Fig. 7:**
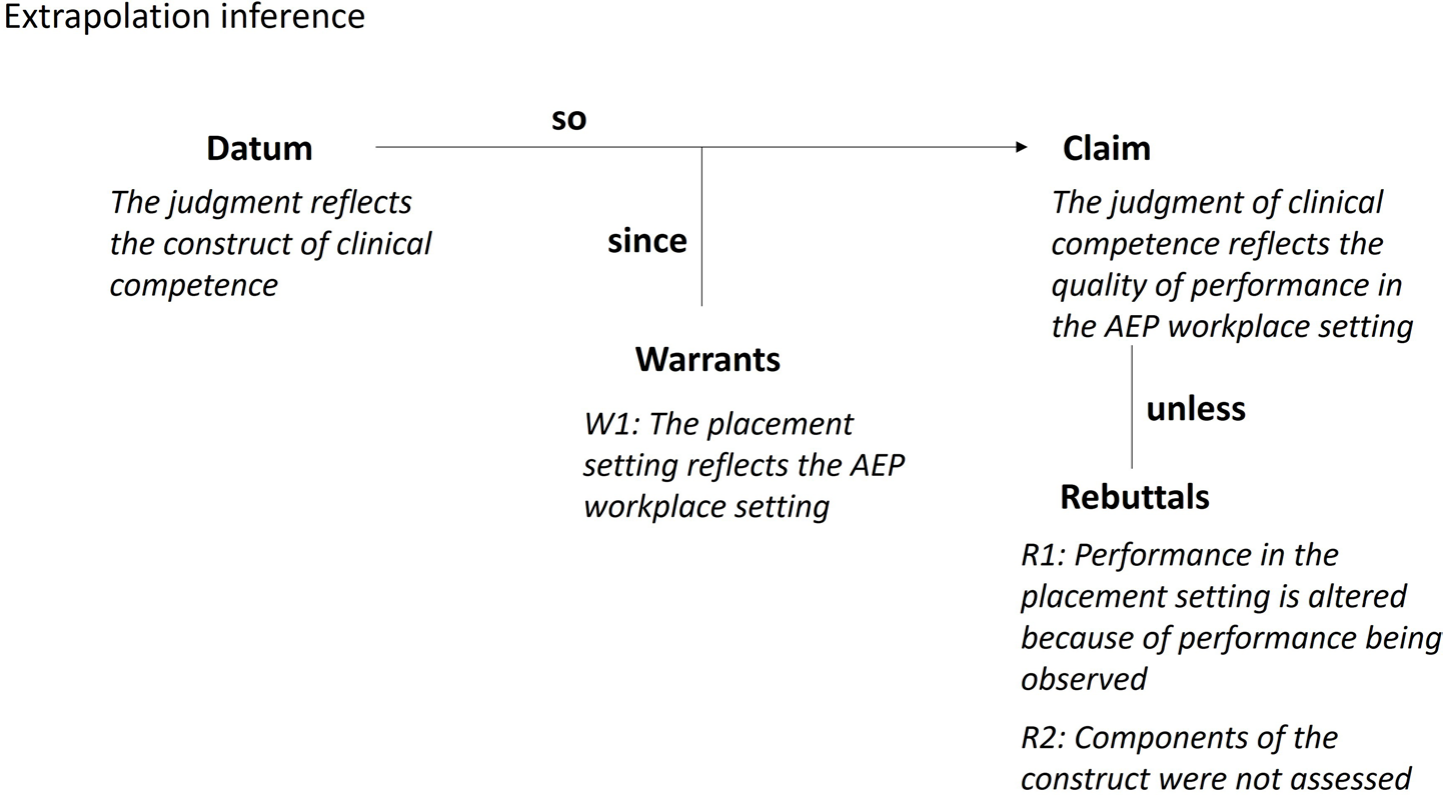
Toulmin diagram for the extrapolation inference

The Toulmin diagram for the extrapolation inference is shown in Fig. 7. The target domain is the real-world AEP professional workplace. The extrapolation inference proposes that the placement educator’s judgment of the student’s clinical competence on the placement reflects the quality of the student’s performance in any AEP professional workplace setting. The warrant supporting this inference is that the placement (activities, environment) during which performance is observed and a judgment made, has sufficient features in common with all environments in which AEP work and the activities they undertake.

The assumptions underlying the warrant include:

- A1: The construct embodies all relevant features of the AEP professional workplace setting.
- A2: The range of activities observed during the placement resemble the range of activities within the AEP professional workplace.
- A3: Judgments made about performance on placement predict performance in the AEP professional workplace.

Potential sources of backing for the warrant include interviews with stakeholders on their views about how strong a proxy the assessment of clinical competence in their placement is for identifying competent performance in a real-life AEP workplace setting. Further backing could be sought by examining stakeholder agreement that a student’s performance on placement reflects likely success if the graduate was in an AEP professional role. As students themselves are stakeholders, another source of backing could include a self-assessment of how confident students feel in their ability to perform in any AEP workplace setting based on their performance on their placements.

#### Utilisation inference

**Fig. 8:**
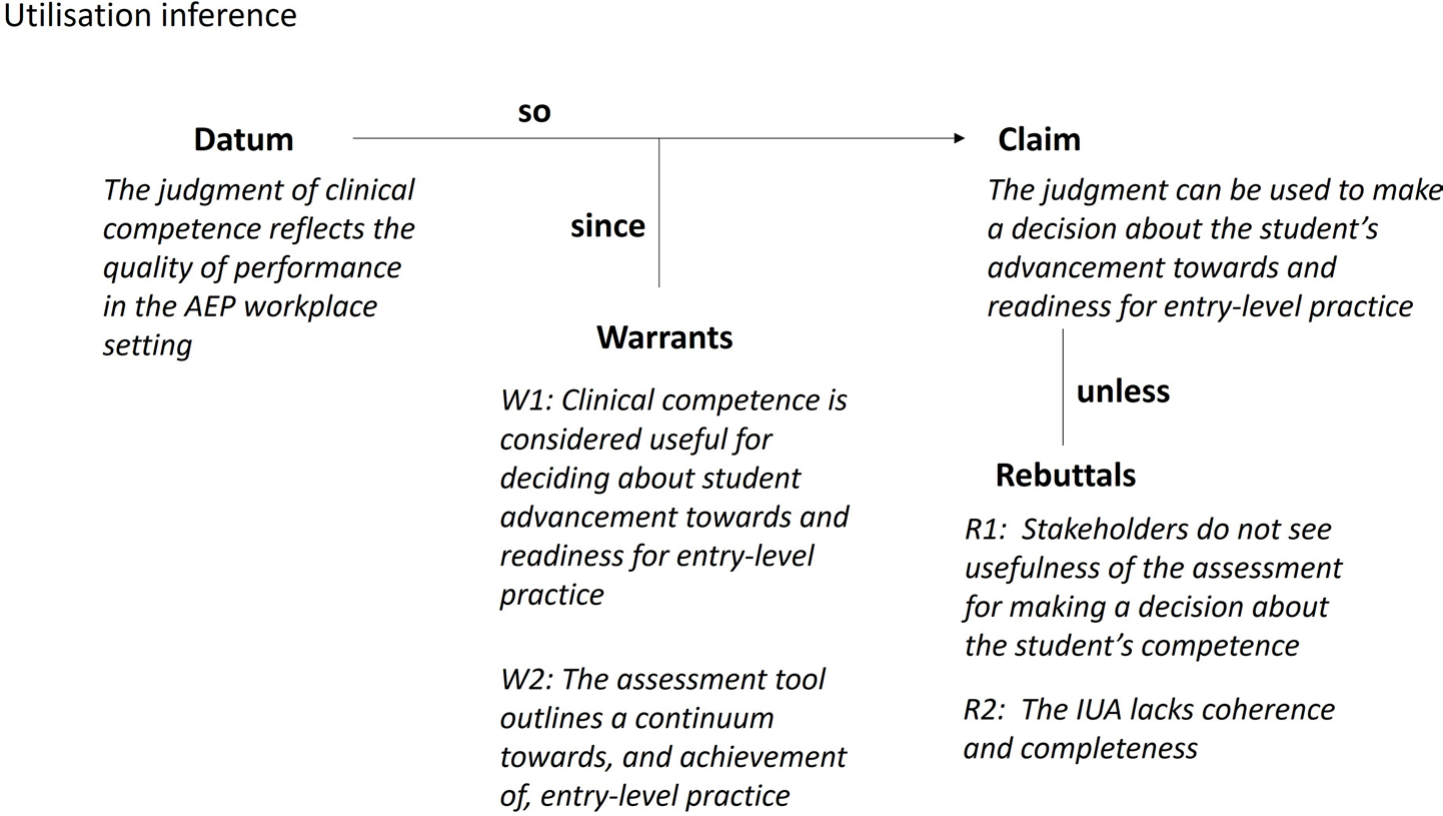
Toulmin diagram for the utilisation inference

The Toulmin diagram for the utilisation inference is shown in Fig. 8. The utilisation inference outlines how the outcome of the assessment (i.e. the placement educator’s judgement of the student’s level of competence) will be used to make a decision. There are two warrants supporting this inference. First, that clinical competence in the AEP workplace setting is considered useful for making a decision about advancement towards, and readiness for entry-level practice. Second, that the assessment tool accurately outlines a continuum towards, and achievement of entry-level practice. The assumptions underlying the warrant include:

- A1: The placement educator can use the VAS in a way that allows students to be differentiated into levels that are needed to make the decision.
- A2: The description of entry level performance on the assessment tool reflects entry-level practice.
- A3: Stakeholders can interpret the assessment outcome and use this to make a decision regarding student progression.
- A4: Stakeholders agree that the assessment outcome is consistent with an overall view about the student’s clinical competence.
- A5: The assessment tool has a positive impact on teaching and learning.

Potential sources of backing for the utilisation inference include a theoretically and empirically supported argument supporting the design of the assessment tool (the scale, the descriptors, the continuum of performance, etc.) and stakeholder feedback about the scale and whether it permits a decision to be made. Evidence could also come from interviews with recent graduates and employers of recent graduates about perceived readiness for entry-level practice as an AEP.

## Lessons learned

This paper presents an IUA tailored for our workplace-based performance assessment tool using the principles of Kane’s approach to argument-based validity. We have provided examples at each inference level of the type of evidence that needs to be collected to support a validity argument for this assessment. While these examples are not exhaustive, they may offer ideas for HPE scholars and educators to use in their local educational context. Our work has led to several insights which we consider useful to share with fellow HPE scholars:

i. One should not be limited to the often used four inferences of scoring, generalisation, extrapolation and utilisation or even the six we have used. Kane’s approach requires the assessment developer to use whatever inferences are necessary to build a transparent, coherent and complete argument for making decisions based on assessment outcomes.
ii. Building the IUA is an iterative process and requires ongoing discussions with your assessment development team. Through these discussions, warrants and assumptions can be challenged and the inferences and links between them critiqued. As a result of our iterative process, the final IUA presented in our worked example is quite different to our first attempt.
iii. We found Toulmin diagrams to be useful for unpacking what is in an inference and identifying linkages in the chain of inferences. This approach helped us appreciate the importance of laying out the claims, warrants and assumptions, a critical step that is often missing, poorly articulated or incomplete in the HPE literature (Kinnear et al., 2022).
iv. Developing the IUA can be a time-consuming and complex undertaking, particularly if using Toulmin’s model of argumentation to lay out the IUA framework. While such an approach has permitted transparency in our worked example, some might find this level of complexity overwhelming or not sustainable in their education context. About the IUA, Kane wrote “… it does not need to be spelled out exhaustively; that would be deadening.” (Kane, 2013, p. 13). At its core, the IUA just needs to be sufficiently detailed to guide evidence collection for the validity argument (Kane, 2013). The level of detail would likely vary depending on the complexity of the assessment and the context.
v. Designing workplace-based performance assessments involves managing a balance between generalisation and extrapolation as both are desirable outcomes but achieving one may negatively impact the other. Kane wrote about this challenge in his 2013 paper, referring to it as the reliability/validity paradox (Kane, 2013, p. 36). In our example, assessment occurs in a highly representative context, the workplace, and therefore extrapolation to “real world” tasks and performance is presumably strong. However, this will need to be balanced against the potential weakness of the generalisability inference due to variation between placement environments and between placement educators’ judgments if the assessment tool design is not effective in mitigating this risk.
vi. The IUA should undergo cycles of refinement as the assessment tool is developed. Kane has acknowledged that, while the IUA should be developed prior to the design of the assessment, it can be further refined as the assessment is developed and limitations become evident (Kane, 2013). In our example, we found that as the assessment tool was being developed, new assumptions came to light. The development phase also provided opportunities to collect potential sources of evidence that could later be used to test the strength and coherence of our IUA. Therefore, in our experience thinking about the IUA both before and during the development of the assessment was helpful.

## Conclusion

This paper presents a worked example of an IUA as it applies to a workplace-based performance assessment. Kane states that argument-based validity is simple (Kane, 2013), but in our experience, this is not necessarily true. Developing an IUA is cognitively demanding. It requires application of principles to a unique assessment situation, as opposed to implementing a templated approach– the IUA is not one-size fits all. Grappling with the process of situating the IUA in the unique context of the assessment in question and providing clear explanations of each inference will strengthen HPE assessment development. A tailored IUA can be critiqued by others, provides a framework for collecting validity evidence and generating a validity argument, and through this transparency, avoids potential for mischief in the assessment validation efforts and subsequent use of assessment outcomes.

## Data Availability

No datasets were generated or analysed during the current study.
